# Efficacy and safety of inetetamab-containing regimens in patients with HER2-positive metastatic breast cancer: a real-world retrospective study in China

**DOI:** 10.3389/fonc.2023.1136380

**Published:** 2023-06-19

**Authors:** Xiaoyu Liu, Peng Zhang, Chao Li, Xiang Song, Zhaoyun Liu, Wenna Shao, Sumei Li, Xinzhao Wang, Zhiyong Yu

**Affiliations:** ^1^ First Clinical Medical College, Shandong University of Traditional Chinese Medicine, Jinan, China; ^2^ Department of General Surgery, Zouping People’s Hospital, Binzhou, China; ^3^ Breast Cancer Center, Shandong Cancer Hospital and Institute, Shandong First Medical University and Shandong Academy of Medical Sciences, Jinan, China; ^4^ College of Traditional Chinese Medicine, Shandong University of Traditional Chinese Medicine, Jinan, China; ^5^ REMEGEN, LTD, Yantai Economic & Technological Development Area, Yantai, China

**Keywords:** breast cancer, inetetamab, human epidermal receptor 2 positive, monoclonal antibody, real-word data

## Abstract

**Background:**

Inetetamab (cipterbin) is an innovative anti-HER2 humanized monoclonal antibody. The efficacy and safety of a combination of inetetamab and vinorelbine in the first-line treatment of human epidermal receptor positive (HER2+) metastatic breast cancer (MBC) have been confirmed. We aimed to investigate real-world data of inetetamab in complex clinical practice.

**Methods:**

We retrospectively reviewed the medical records of patients who received inetetamab as a salvage treatment at any line setting from July 2020 to June 2022. The main endpoint was progression‐free survival (PFS).

**Results:**

A total of 64 patients were included in this analysis. The median progression‐free survival (mPFS) was 5.6 (4.6–6.6) months. Of the patients, 62.5% received two or more lines of therapy before treatment with inetetamab. The most common chemotherapy and anti-HER2 regimens combined with inetetamab were vinorelbine (60.9%) and pyrotinib (62.5%), respectively. Patients treated with inetetamab plus pyrotinib plus vinorelbine benefited the most (p=0.048), with the mPFS of 9.3 (3.1–15.5) months and an objective response rate of 35.5%. For patients with pyrotinib pretreatment, inetetamab plus vinorelbine plus pyrotinib agents resulted in mPFS of 10.3 (5.2–15.4) months. Regimens (inetetamab plus vinorelbine plus pyrotinib vs. other therapeutic agents) and visceral metastases (yes vs. no) were independent predictors of PFS. Patients with visceral metastases treated with inetetamab plus vinorelbine plus pyrotinib had a mPFS of 6.1(5.1–7.1) months. The toxicity of inetetamab was tolerable, with the most common grade 3/4 adverse event being leukopenia (4.7%).

**Conclusions:**

HER2+ MBC patients pretreated with multiple-line therapies still respond to inetetamab-based treatment. Inetetamab combined with vinorelbine and pyrotinib may be the most effective treatment regimen, with a controllable and tolerable safety profile.

## 1 Introduction

Breast cancer(BC) is the most common cancer and a leading cause of death among women worldwide (1). There are three major breast cancer subtypes: hormone receptor positive(estrogen receptor (ER) positive or progesterone receptor(PR) positive), human epidermal receptor 2 (HER2) positive (HER2+) and triple negative breast cancer (ER negative, PR negative, HER2 negative) (2). HER2 is a transmembrane receptor tyrosine kinase in the epidermal growth factor receptor family that is amplified or overexpressed in approximately 20% of breast cancers, and is associated with poor prognosis in the absence of systemic therapy (3). HER2+ ductal tumors are associated with the presence of calcifications, as well as high tumor grade and increased likelihood of spread to the lymph nodes (4, 5). Without the development and widespread use of anti‐HER2‐targeted drugs, HER2+ BC is an aggressive disease and has poor prognosis (6). Although huge progresses have been achieved in the last few years in understanding and treating HER2+ breast cancer, they remain a disproportionate health burden to patients and huge unmet need (7). Especially, HER2+ metastatic breast cancer(MBC) remains incurable, and novel treatment options are needed. Many anti‐HER2‐targeted drugs have been applied successfully in clinical or currently under review in recent years. Antibody–drug conjugates(ADC) drugs are on rise and provides novel therapeutic advancements in the management of HER2+ MBC (8). But in China, they are expensive and not included in medical insurance which limited their usage.

The innovative drug of recombinant anti-HER2 humanized monoclonal antibody (inetetamab, Cipterbin) for injection independently researched and developed in China is a non-biological analog drug produced by Sansheng Guojian Pharmaceutical (Shanghai) Co., Ltd. (formerly CITIC Guojian Pharmaceutical Co., Ltd.) and approved by the State Food and Drug Administration of China for clinical research on July 2, 2004 (Approval No. 2004L02352). Inetetamab is a monoclonal antibody binding to domain IV of HER2 receptor. The Fab domain of inetetamab is identical with trastuzumab, but whose amino acid sequence at positions 359 (D359, aspartic acid) and 361 (L361, leucine) is different from trastuzumab (E359 (glutamate) and M361 (methionine), respectively) in the constant region of the heavy chain of the Fc domain (9). Previous study confirmed the significant efficacy and good safety of the combination of cipterbin and vinorelbine in the first-line treatment of HER2 positive advanced breast cancer patients who had not received anti-HER2-targeted therapy after previous taxus treatment (10).

Based on the current situation of clinical treatment and needs, we conducted a retrospective study to fill a knowledge gap by investigating the efficacy of inetetamab for HER2+ recurrent and metastatic breast cancer patients pretreated with multi-lines treatment.

## 2 Material and methods

### 2.1 Subjects and study design

This retrospective, single-center study enrolled patients with HER2+ MBC treated with inetetamab at Shandong Frist Medical University and Shandong Academy of Medical Sciences between July 2020 and June 2022. The Ethics Committee and Institutional Review Board of Shandong First Medical University and Shandong Academy of Medical Sciences approved this study (SDTHEC2022012020). All investigations were conducted in accordance with the Declaration of Helsinki.

### 2.2 Patients

The inclusion criteria for participants were as follows: female sex, age ≥18 years, histologically or cytologically confirmed MBC with documentation of HER2 overexpression, prior trastuzumab therapy with or without other HER2-targeted treatment, at least one cycle of inetetamab, and complete medical records. The exclusion criteria were non-measurable or non-evaluable lesions and those lost to follow-up. There were no limits to the number of prior cytotoxic regimens for metastatic diseases. The last follow-up was conducted in November 2022. Until the last follow-up date, patients who were lost to follow-up were considered as censored data. All data were retrospectively collected from medical records and laboratory results. Patients or their family members (for patients who already died at the study initiation) provided signed informed consent or oral agreement with tape recording.

### 2.3 Assessments

The characteristics of the patients at the time of initial diagnosis (including age, ECOG performance status, and menstrual status), tumor characteristics (including tumor size, lymph node involvement, grade, histology, and receptor status), treatment regimen in the (neo)adjuvant and metastatic settings (including chemotherapy, anti-HER2, endocrine regimen, surgery, radiotherapy, dose reductions or delays, etc.) were extracted from electronic medical records. Hormone receptor (HR) was defined as estrogen receptor (ER) and/or progesterone receptor (PR) positivity (ER and PR were determined by at least 10% of positively stained nuclei). HER2 positivity was defined as an immunohistochemistry(IHC) score of 3+ or 2+ together with HER2 gene amplification verified by fluorescence *in situ* hybridization(FISH+). Disease-free interval (DFI) was defined as the time from surgery to diagnosis of metastasis.

Clinical response was evaluated using computed tomography, magnetic resonance imaging, and physical examination according to the Response Evaluation Criteria in Solid Tumors, version 1.1. The main endpoint was progression-free survival (PFS), defined as the time from treatment initiation until disease progression or death. Other endpoints included the objective response rate (ORR), clinical benefit rate (CBR), and safety. ORR was defined as the proportion of patients who achieved a complete response (CR) or partial response (PR). CBR was defined as the proportion of patients who achieved CR, PR, or stable disease (SD). Adverse events (AEs) were graded based on the National Cancer Institute Common Terminology Criteria for AEs, version 4.0.

### 2.4 Statistical analyses

The median (range) or percentage of patients was used to represent the clinicopathological characteristics. Continuous variables were analyzed by One-way ANOVA. Categorical variables were assessed by the Pearson’s chi-squared test or Fisher’s exact test. The Kaplan–Meier method was used to estimate PFS. Additionally, Cox univariable model was employed to assess the covariate effects on PFS, and then Cox multivariate models were used to assess the factors with relative significant p-values(p ≤ 0.1) in univariate analysis to PFS with hazard ratios(HR.) and corresponding 95% confidence intervals (CIs). Statistical significance for all analyses was set at p<0.05. GraphPad Prism 9.3.1 software was used to perform all statistical analyses.

## 3 Results

### 3.1 Patients and treatments

**Figure 1:**
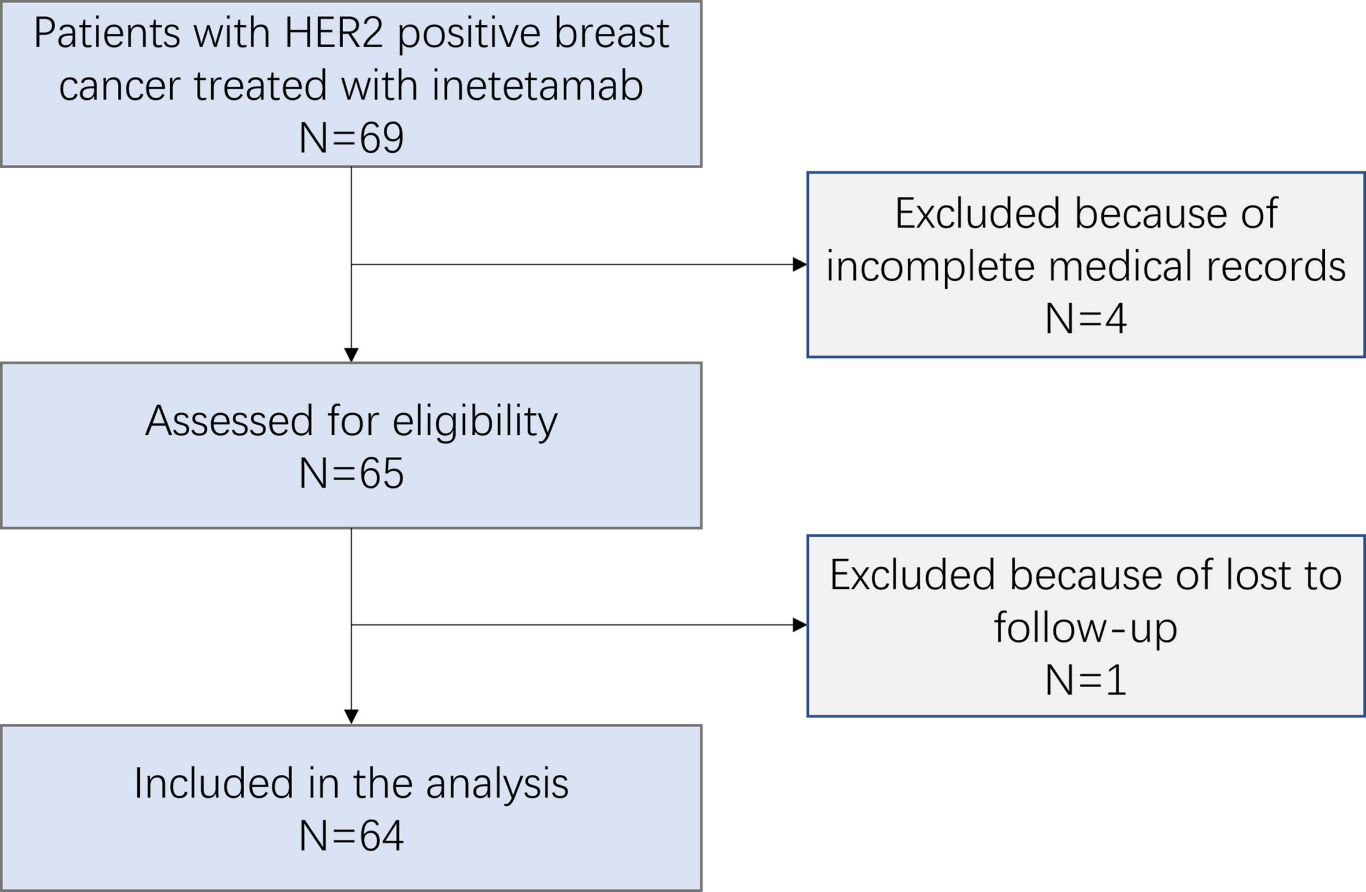
Patient’s profile.

A total of 69 patients with HER2+ MBC treated with inetetamab were recruited. After considering the exclusion criteria, 64 (92.8%) patients were included in the study (**Figure 1**).

The baseline characteristics are presented in **Table 1**. The median age of the patients at diagnosis was 46 years (range: 27–67 years), 54 (84.4%) underwent surgery, 14 patients (21.9%) had stage IV BC as their first diagnosis. Moreover, 53.1% of the patients had more than two metastatic sites, with the four most common metastatic sites being the lymph node (48.4%), local sites (35.9%), bone (32.8%), and liver (32.8%). Half of the patients had visceral metastases, whereas 13 (20.3%) had brain metastases. All patients had been exposed to anti-HER2 therapy, with 64.1% prescribed pyrotinib and 18.8% with lapatinib. More than four-fifths of the patients received trastuzumab during salvage treatment. Furthermore, 62.5% of patients received two or more lines of systemic therapy before inetetamab. These results suggest that, in a real-world setting, patients receiving inetetamab are more likely to be heavily pretreated.

**Table 1:** Baseline characteristics of patients.

Characteristics	Patients, No (%) N = 64	
Age, median (range years)	46 (27-67)	
Menstrual status		
pre	38 (59.4)
post	26 (40.6)
ECOG performance status		
0-1	52 (81.3)
≥2	12 (18.8)
Pathological type		
Invasive ductal cancer	62 (96.9)
Invasive lobular cancer	2 (3.1)
HER2 expression		
IHC2+ and FISH+	13 (20.3)
IHC3+	51 (79.7)
HR status at metastatic setting		
Positive	29 (45.3)
Negative	35 (54.7)
Surgery		
No	10 (15.6)
Yes	54 (84.4)
Radiotherapy		
No	35 (54.7)
Yes	29 (45.3)
Endocrine therapy		
No	41 (64.1)
Yes	23 (35.9)
DFI (month)		
≤12	16 (25.0)
>12	34 (53.1)
*De novo* IV stage	14 (21.9)
Previous trastuzumab treatment		
Neoadjuvant setting	5 (7.8)
Adjuvant setting	22 (34.4)
Metastatic setting	52 (81.3)
Previous anti‐HER2 drugs		
Pyrotinib	41 (64.1)
Pertuzumab	10 (15.6)
TDM-1	2 (3.1)
Aptinib	2 (3.1)
Lapatinib	12 (18.8)
Anlotinib	1 (1.6)
Number of sites in primary recurrence		
1	34 (53.1)
>1	30 (46.9)
Lines of inetetamab in metastatic setting		
1	3 (4.7)
2	21 (32.8)
≥3	40 (62.5)
Number of sites before inetetamab		
1	21 (32.8)
2	9 (14.1)
≥3	34 (53.1)
Metastatic sites before inetetamab		
Local sites	23 (35.9)
Lymph node	31 (48.4)
Bone	21 (32.8)
Brain	13 (20.3)
Lung	15 (23.4)
Liver	21 (32.8)
Others	11 (17.2)
Visceral metastases		
Yes	32 (50.0)
No	32 (50.0)

### 3.2 Treatment administration

The treatment regimens are shown in **Table 2**. Most patients were treated with inetetamab in combination with chemotherapy and/or other HER2-targeted therapies. The most common chemotherapy regimens were vinorelbine (n = 39, 60.9%) and abraxane (n = 15, 23.4%). Pyrotinib and inetetamab in combination were administered to 40 (62.5%) patients. Meanwhile, three (3.1%) patients received inetetamab and brain radiotherapy but did not receive any other anti-cancer drugs.

**Table 2:** Treatment administration.

Treatment administration		Patients, No (%) N = 64
Combined regimens with inetetamab		
Vinorelbine		39 (60.9)
Abraxane		15 (23.4)
Other		7 (10.9)
No		3 (3.1)
Target regimens with inetetamab		
Pyrotinib		40 (62.5)
Pertuzumab		6 (9.4)
Alone		18 (28.1)

### 3.3 Treatment efficacy in overall patients

**Figure 2:**
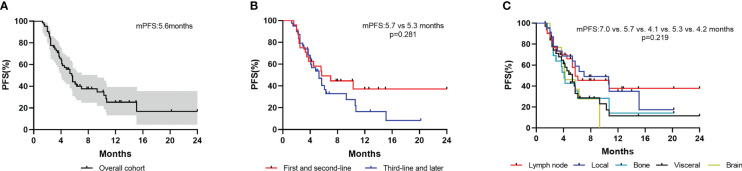
Kaplan–Meier curves of PFS for patients. **(A)** Overall cohort; **(B)** Patients stratified by treatment lines; **(C)** Patients with different metastatic sites.

All patients were evaluated for PFS. The median follow-up time was 14.3(12.7–15.9) months. The median progression-free survival (mPFS) was 5.6 (4.6–6.6) months and the ORR was 26.6% (**Figure 2A**).

Patients who received inetetamab‐based therapy as first and later lines of metastatic treatment had a median PFS of 5.7 (1.9–9.5) and 5.3 (3.8–6.8) months, respectively (**Figure 2B**). Thirty-two patients with visceral metastases showed a median PFS of 5.3 (3.9–6.7) months. A total of 31 patients with lymph node metastases, 23 patients with local metastases, 21 patients with bone metastases and 13 patients with brain metastases had median PFS of 5.7 (0.5–10.9) months, 7.0 (0.8–13.2) months, 4.1(3.4–4.8) months and 4.2 (2.0–6.4) months, respectively (**Figure 2C**).

**Figure 3:**
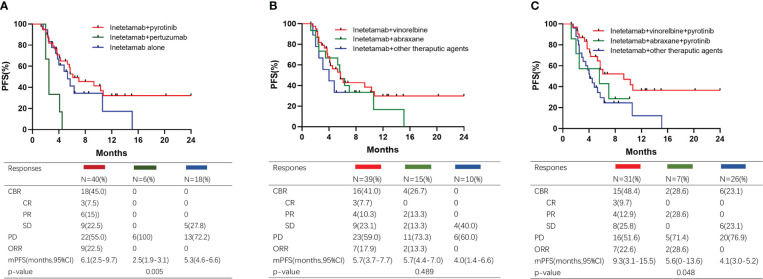
Kaplan–Meier curves of PFS and responses for different treatment. **(A)** Patients treated with different dual anti-HER2 therapy; **(B)** Patients treated with different chemotherapy; **(C)** Patients treated with different combined regimens.

To determine the best combination for inetetamab, we firstly investigated the anti-HER2 treatment in the overall cohort. Baseline characteristics were analyzed in **Supplementary Tables 1-3**. The median PFSs among inetetamab plus pyrotinib, inetetamab plus pertuzumab and inetetamab alone were 6.1 (2.5–9.7) months, 2.5 (1.9–3.1) months, and 5.3 (4.6–6.6) months, respectively. Inetetamab plus pyrotinib was the best combination among the three groups (p=0.005) (**Figure 3A**). However, the age of patients treated with inetetamab plus pertuzumab is relatively old than other two cohorts(p=0.001) and 6 patients(100%) received at least 3 lines of rescue treatment(p=0.016), which led to the unbalanced baseline characteristics among three cohorts. As vinorelbine and abraxane were the most common combined cytotoxic drugs, we compared the PFS of different chemotherapies. The median PFSs among inetetamab plus vinorelbine, inetetamab plus abraxane and inetetamab plus other therapeutic agents were 5.7 (3.7-7.7) months, 5.7 (4.4–7.0) months, and 4.0 (1.4–6.6) months, respectively (**Figure 3B**). Furthermore, we compared the efficacy of the combination treatments. The median PFS of inetetamab plus pyrotinib plus vinorelbine was 9.3 (3.1–15.5) months; inetetamab plus pyrotinib plus abraxane, 5.6(0-13.6) months; and inetetamab plus other therapeutic agents, 4.1 (3.0–5.2) months. There were statistically significant differences among the three groups (p = 0.048) (**Figure 3C**). These findings indicate that inetetamab plus pyrotinib plus vinorelbine may be the most effective inetetamab-based regimen. Thirty-one (48.4%) patients received inetetamab plus pyrotinib plus vinorelbine. The subgroup of patients achieved an ORR of 35.5% and CBR of 48.4%, with CR achieved in three patients, PR achieved in eight patients, and SD achieved in four patients (**Figure 3C**).

### 3.4 Efficacy of inetetamab-based therapy in certain drugs pretreated patients

**Figure 4:**
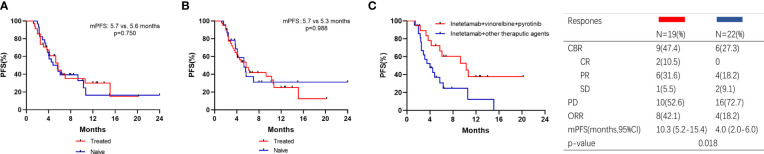
Kaplan–Meier curves of PFS for patients and responses. **(A)** Patients with vinorelbine‐treated or vinorelbine‐naive; **(B)** Patients with pyrotinib‐treated or pyrotinib‐naive; **(C)** Patients who were pyrotinib‐treated received inetetamab + vinorelbine + pyrotinib or other therapeutic agents.

Thirty-five patients received vinorelbine before inetetamab-based therapy. The mPFS of patients with versus without vinorelbine pretreatment was 5.7 (4.0–7.4) months versus 5.6 (3.5–7.7) months, respectively (p=0.750) (**Figure 4A**). Forty-one patients received pyrotinib before inetetamab‐based therapy. The mPFS of patients with versus without pyrotinib pretreatment was 5.7 (3.5–7.9) months versus 5.3 (3.3–7.3) months, respectively (p=0.988) (**Figure 4B**). These results indicate that the medication history of vinorelbine and/or pyrotinib had no influence on the efficacy of the drug.

We also analyzed the PFS of the patients pretreated with pyrotinib (**Figure 4C**). A total of 41 patients were included in this subgroup analysis, with an ORR of 29.3%. Two patients achieved CR and 10 patients achieved PR. Patients exposed to inetetamab plus vinorelbine plus pyrotinib agent had significantly longer PFS (10.3 (5.2–15.4) months) than those exposed to other therapeutic agents (4.0 (2.0–6.0) months) (p=0.018).

### 3.5 Efficacy in patients with visceral metastasis

The univariate analysis indicated that age group (<40 vs. ≥40 years), menstrual status (pre vs. post), hormone receptor status (negative vs. positive), and regimens (inetetamab plus vinorelbine plus pyrotinib vs. other therapeutic agents) were correlated with PFS (p<0.05). Next, we constructed a multivariate model with the above factors, ECOG performance status(0-1 vs ≥2) and visceral metastasis (yes vs. no) as covariates for PFS (**Table 3**). After adjustment, Cox multivariate regression analysis showed that the regimens (inetetamab plus vinorelbine plus pyrotinib vs. other therapeutic agents) and visceral metastasis (yes vs. no) were independent predictors of PFS (**Table 3**).

**Table 3:** Univariate and multivariate analysis of factors associated with progression‐free survival.

Characteristic	HR. (95% CI)	Univariate analysis p-value	HR. (95% CI)	Multivariate analysis p-value
Age group (<40 vs. ≥40)	0.433 (0.224-0.839)	0.013	0.528 (0.238-1.174)	0.117
ECOG performance status (0-1 vs ≥2)	0.419 (0.162-1.088)	0.074	0.440 (0.150-1.295)	0.136
Menstrual status (pre vs. post)	0.454 (0.230-0.896)	0.023	1.156 (0.468-2.860)	0.753
Hormone receptor status (negative vs. positive)	1.982 (1.064-3.692)	0.031	1.426 (0.704-2.885)	0.324
DFI (≤12 months vs.>12 months vs. *de novo* IV stage)	1.375 (0.564-3.353)	0.484	N/A*	N/A
Number of sites in primary recurrence (1 vs. >1)	1.350 (0.729-2.498)	0.340	N/A	N/A
Number of metastatic sites before inetetamab (1 vs. ≥2)	0.838 (0.447-1.573)	0.583	N/A	N/A
Brain metastasis (no vs. yes)	1.516 (0.731-3.145)	0.263	N/A	N/A
Visceral metastasis (no vs. yes)	1.735 (0.928-3.244)	0.084	2.444 (1.095-5.457)	0.029
Regimens (other therapeutic agents vs. inetetamab + vinorelbine + pyrotinib)	2.167 (1.140-4.119)	0.018	3.543 (1.680-7.471)	0.001

*N/A, Not applicable.

**Figure 5:**
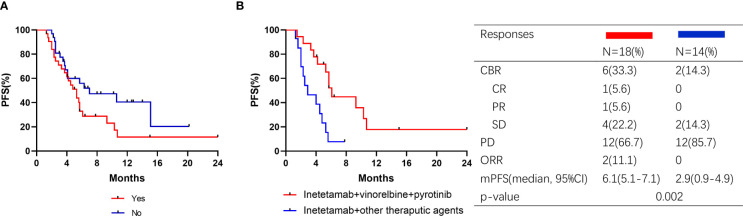
Kaplan–Meier curves of PFS for patients and responses in subgroup. **(A)** Patients with visceral metastasis or not; **(B)** Visceral metastasis patients treated with pyrotinib or pyrotinib + trastuzumab.

Thirty-two patients (50.0%) exhibited visceral metastasis. Patients with and without visceral metastases had PFS times of 5.3 months and 7.0 months, respectively (**Figure 5A**). Of the 32 patients, 18 received inetetamab plus vinorelbine plus pyrotinib treatment, with an ORR of 27.8% and a CBR of 33.3%. One patient achieved CR, four achieved PR, and one achieved SD. The median PFS was significantly different for patients who underwent inetetamab plus vinorelbine plus pyrotinib or other therapeutic agents (6.1 (5.1–7.1) vs. 2.9-(0.9–4.9) months, p=0.002; **Figure 5B**).

### 3.6 Safety assessment

The safety assessments of etamab-based therapy are listed in **Table 4**. After the initial etamab-based therapy, 10 (15.6%) patients in the inetetamab group experienced a dose reduction, and two (3.1%) patients interrupted the treatment. The most common grade 3/4 AEs were leukopenia (4.7%) and neutropenia (3.1%). No treatment-related deaths were reported. Overall, the results show that the safety of etamab-based therapy is controllable and tolerable.

**Table 4:** Safety assessment.

Safety assessment		Patients, No (%) N = 64
Dose reduction of inetetamab treatment due to AEs		10 (15.6)
Interruption of inetetamab treatment due to AEs		2 (3.1)
Adverse events (grade 3/4)		
Neutropenia		2 (3.1)
Leukopenia		3 (4.7)
Thrombocytopenia		1 (1.6)
Anemia		1 (1.6)
Aminotransferase increased		1 (1.6)
Hand-foot syndrome		1 (1.6)

## 4 Discussion

This study revealed the real-world clinical practice of inetetamab in HER2+ MBC patients after trastuzumab-based treatment. Previously, the efficacy and safety of inetetamab in combination with chemotherapy as first-line treatment of HER2+ MBC was evaluated (9). But the above study of inetetamab was designed for patients who did not receive any anti-HER2 drugs. Therefore, the role of inetetamab in more heavily treated patients needs further study. To the best of our knowledge, this is the first investigation of the effectiveness of inetetamab in HER2+ MBC patients pretreated with multiline anti-HER2 treatment. Our cohort represented the general population of patients with HER2+ MBC who were usually heavily treated with multiple anti-HER2 agents. Yet, our cohort included a low percentage of patients receiving TDM1(3.1%) and no patient receiving new drugs such as Trastuzumab Deruxtecan (T-DXd) or Tucatinib, which limited our research.

The combination of inetetamab, pyrotinib and vinorelbine, as evidence-based, trustworthy and promising drugs, play a synergistic role in efficacy. Pyrotinib, a small-molecule irreversible tyrosine kinase inhibitor(TKI), has attracted much attention due to its unique properties in recent years. According to the National Comprehensive Cancer Network guidelines, pyrotinib is a valid treatment option. A number of reports have verified the therapeutic efficacy of pyrotinib in HER2+ MBC. Several multicenter analyses showed that pyrotinib treatment led to a mPFS time of about 8 months (11, 12) the ORR of 17.1% in two or later line therapy (13). Besides, the clinical benefits and safety of dual HER2 blockade by anti-HER2 monoclonal antibody plus TKI for patients that had progressed during trastuzumab-based treatment regimens were confirmed (14–16). Thus, inetetamab, as an identical monoclonal antibody with trastuzumab, combined with pyrotinib led to a satisfactory efficacy. On the other hand, vinorelbine is a semi-synthetic, antimitotic, microtubule destabilizing drug that has been shown to be effective and well-tolerated for the treatment of MBC (17). It is noteworthy that compared with other chemotherapy drugs, the combined index CI of vinorelbine and trastuzumab was only 0.34 (18–20). It is suggested that the combination of vinorelbine and anti-HER2 monoclonal antibody has the best synergistic effect. In first-line treatment, the combination of vinorelbine with trastuzumab and pertuzumab reached the mPFS of 14.2 months, indicated that vinorelbine plus dual anti-HER2 therapy showed successful anti-tumor activity and few adverse effects (21, 22).. A retrospective study reported that the mPFS of patients treated with metronomic vinorelbine and triweekly trastuzumab was 8.9 months (23). Two multicenter retrospective studies showed pyrotinib plus vinorelbine therapy had promising efficacy and tolerable toxicity in HER2+ MBC, with mPFS of 7.8 and 8.3 months, respectively (24, 25). In addition, pyrotinib combined with vinorelbine in HER2+ MBC was effective regardless of resistant status of trastuzumab (24, 25).

Considering that inetetamab is similar to trastuzumab (9), the combination of inetetamab and trastuzumab should have a good efficacy in metastatic setting. But the small sample data resulted in some analysis biases and the inability to conduct depth analysis. Despite the combination of inetetamab plus pyrotinib plus vinorelbine showed satisfactory outcomes, which was comparable to the mPFS of 9.6 months in the EMILIA study (26), there is a significant gap compared to T-DXd(mPFS=28.8 months) according to the updated results from DESTINY-Breast03 trial (27). Notwithstanding, high prices of TDM1 and T-DXd results in limitations in the ability to use in clinical practice. Whereas, inetetamab plus pyrotinib plus vinorelbine can be considered as an alternative treatment option.

Brain and visceral metastases have poor prognosis and limit treatment for HER2+ MBC (3, 28). For patients with visceral metastasis, the outcomes of the combination regimen are inferior to that reported for pyrotinib-based regimens in the previous multicenter retrospective study (24, 29–32). The reason might be in our study, patients were less sensitive to anti-HER2 treatment after multi-lines treatment, especially after pyrotinib-based treatment. Despite mounting evidence verified that pyrotinib-based combination therapy was efficient to treat HER2+ brain metastasis (11, 33–37), brain metastasis was not a significant factor affecting the efficacy of inetetamab in our study and the recruited patients with brain metastasis was too little for further analysis.

In terms of toxicity, the published results of the large clinical trials indicated that there were no significant change in grades and incidences of AEs, showing that inetetamab and trastuzumab are equivalently safe (9). Inetetamab-based therapy was also tolerated in our study. Yet, the medical records might omit important information about AEs even though we have thoroughly reviewed the patient’s examination results and medical records, which resulted in deviations in our results.

In conclusion, major populations of HER2+ MBC patients previously treated with multiple anti‐HER2 therapies including trastuzumab still responded to inetetamab‐based treatment in clinical practice. Inetetamab combined vinorelbine and pyrotinib might be the most effective inetetamab-based regimen. And the safety of inetetamab was controllable and tolerable. Notwithstanding the efficacy and safety of clinical trials are applicable for two or later‐line inetetamab‐based therapy remains questionable, our study of a series of patients provides real‐world data to further explore inetetamab-based treatment patterns and more experience outside the clinical trials for clinicians in treating general HER2+ MBC patients.

## Data availability statement

The raw data supporting the conclusions of this article will be made available by the authors, without undue reservation.

## Ethics statement

The studies involving human participants were reviewed and approved by The Ethics Committee and Institutional Review Board of Shandong First Medical University and Shandong Academy of Medical Sciences (SDTHEC2022012020). The patients/participants provided their written informed consent to participate in this study.

## Author contributions

XL and PZ collected the data. XL analyzed data and wrote the manuscript. CL, XS, ZL, WS and SL revised the manuscript. ZY and XW played a role in developing the idea. All authors contributed to manuscript revision, read, and approved the submitted version.
