# Supplementary material for: Efficacy and safety of inetetamab-containing regimens in patients with HER2-positive metastatic breast cancer: a real-world retrospective study in China
Source: Front Oncol. 2023 Jun 19;13:1136380. doi: 10.3389/fonc.2023.1136380 (PMC10316697; doi:10.3389/fonc.2023.1136380)
Supplement: Supplementary file 1 [file Table_1.docx]

**Supplementary Table**

Supplementary Table 1 Patients treated with different dual anti-HER2 therapy

| Characteristics | | Patients, No (%) | | | p-value |
| --- | --- | --- | --- | --- | --- |
|  |  | Inetetamab+pyrotinib, N=40(62.5) | Inetetamab+pertuzumab, N=6(9.4) | Inetetamab alone,  N=18(28.1) |  |
| Age, median (range years) | | 46(27-67) | 52(46-53) | 40(29-53) | 0.001 |
| Menstrual status | | | | | |
|  | pre | 22(55.0) | 2(33.3) | 14(77.8) | 0.127 |
|  | post | 18(45.0) | 4(66.7) | 4(22.2) |  |
| ECOG performance status | | | | | |
|  | 0-1 | 32(80.0) | 6(100) | 14(77.8) | 0.705 |
|  | ≥2 | 8(20.0) | 0(0) | 4(22.2) |  |
| Pathological type | | | | | |
|  | Invasive ductal cancer | 40(100) | 5(83.3) | 17(94.4) | 0.061 |
|  | Invasive lobular cancer | 0(0) | 1(16.7) | 1(5.6) |  |
| HER2 expression | | | | | |
|  | IHC2+ and FISH+ | 8(20.0) | 1(16.7) | 4(22.2) | 1.000 |
|  | IHC3+ | 32(80.0) | 5(83.3) | 14(77.8) |  |
| HR status at metastatic setting | | | | | |
|  | Positive | 18(45.0) | 3(50.0) | 8(44.4) | 1.000 |
|  | Negative | 22(55.0) | 3(50.0) | 10(55.6) |  |
| Surgery | | | | | |
|  | No | 6(15.0) | 0(0) | 4(22.2) | 0.505 |
|  | Yes | 34(85.0) | 6(100) | 14(77.8) |  |
| Radiotherapy | | | | | |
|  | No | 22(55.0) | 4(66.7) | 9(50.0) | 0.744 |
|  | Yes | 18(45.0) | 2(33.3) | 9(50.0) |  |
| Endocrine therapy | | | | | |
|  | No | 24(60.0) | 3(50.0) | 14(77.8) | 0.320 |
|  | Yes | 16(40.0) | 3(50.0) | 4(22.2) |  |
| DFI (month) | | | | | |
|  | ≤12 | 12(30.0) | 2(33.3) | 2(11.1) | 0.235 |
|  | >12 | 20(50.0) | 4(66.7) | 9(50.0) |  |
|  | De novo IV stage | 8(20.0) | 0(0) | 7(38.9) |  |
| Previous trastuzumab treatment | | | | | |
|  | Neoadjuvant setting | 5(12.5) | 0(0) | 0(0) | 0.101 |
|  | Adjuvant setting | 19(47.5) | 1(16.7) | 2(11.1) |  |
|  | Metastatic setting | 30(75.0) | 6(100) | 16(88.9) |  |
| Previous anti‐HER2 drugs | | | | | |
|  | Pyrotinib | 23(57.5) | 4(66.7) | 14(77.8) | 0.317 |
|  | Pertuzumab | 7(17.5) | 2(33.3) | 1(5.6) |  |
|  | TDM-1 | 1(2.5) | 0(0) | 1(5.6) |  |
|  | Aptinib | 1(2.5) | 0(0) | 1(5.6) |  |
|  | Lapatinib | 8(20.0) | 3(50.0) | 1(5.6) |  |
|  | Anlotinib | 0(0) | 0(0) | 1(5.6) |  |
| Number of sites in primary recurrence | | | | | |
|  | 1 | 24(60.0) | 2(33.3) | 8(44.4) | 0.379 |
|  | >1 | 16(40.0) | 4(66.7) | 10(55.6) |  |
| Lines of inetetamab in metastatic setting | | | | | |
|  | 1 | 3(7.5) | 0(0) | 0(0) | 0.016 |
|  | 2 | 10(25.0) | 0(0) | 11(61.1) |  |
|  | ≥3 | 27(67.5) | 6(100) | 7(38.9) |  |
| Number of sites before inetetamab | | | | | |
|  | 1 | 15(37.5) | 0(0) | 6(33.3) | 0.096 |
|  | 2 | 8(20.0) | 0(0) | 1(5.6) |  |
|  | ≥3 | 17(42.5) | 6(100) | 11(61.1) |  |
| Metastatic sites before inetetamab | | | | | |
|  | Local sites | 15(37.5) | 1(16.7) | 7(38.9) | 0.889 |
|  | Lymph node | 19(47.5) | 3(50.0) | 9(50.0) |  |
|  | Bone | 10(25.0) | 4(66.7) | 7(38.9) |  |
|  | Brain | 7(17.5) | 2(33.3) | 4(22.2) |  |
|  | Lung | 11(27.5) | 1(16.7) | 3(16.7) |  |
|  | Liver | 16(40.0) | 1(16.7) | 4(22.2) |  |
|  | Others | 7(17.5) | 1(16.7) | 3(16.7) |  |
| Visceral metastases | | | | | |
|  | Yes | 23(57.5) | 2(33.3) | 7(38.9) | 0.379 |
|  | No | 17(42.5) | 4(66.7) | 11(61.1) |  |

Supplementary Table 2 Patients treated with different chemotherapy

| Characteristics | | Patients, No (%) | | | p-value |
| --- | --- | --- | --- | --- | --- |
|  |  | Inetetamab+ vinorelbine, N=39 (60.9) | Inetetamab+abraxane, N= 15(23.4) | Inetetamab+other therapeutic agents,  N=10 (15.6) |  |
| Age, median (range years) | | 46(27-58) | 49(34-64) | 43(40-67) | 0.467 |
| Menstrual status | | | | | |
|  | pre | 24(64.5) | 8(53.3) | 6(60.0) | 0.934 |
|  | post | 15(23.4) | 7(46.7) | 4(40.0) |  |
| ECOG performance status | | | | | |
|  | 0-1 | 35(89.7) | 10(66.7) | 7(70.0) | 0.093 |
|  | ≥2 | 4(10.3) | 5(33.3) | 3(30.0) |  |
| Pathological type | | | | | |
|  | Invasive ductal cancer | 38(97.4) | 14(93.3) | 10(100.0) | 0.632 |
|  | Invasive lobular cancer | 1(2.6) | 1(6.7) | 0(0) |  |
| HER2 expression | | | | | |
|  | IHC2+ and FISH+ | 8(20.5) | 2(13.3) | 3(30.0) | 0.555 |
|  | IHC3+ | 31(79.5) | 13(86.7) | 7(70.0) |  |
| HR status at metastatic setting | | | | | |
|  | Positive | 18(46.2) | 7(46.7) | 4(40.0) | 1.000 |
|  | Negative | 21(53.8) | 8(53.3) | 6(60.0) |  |
| Surgery | | | | | |
|  | No | 5(12.8) | 4(26.7) | 1(10.0) | 0.486 |
|  | Yes | 34(87.2) | 11(73.3) | 9(90.0) |  |
| Radiotherapy | | | | | |
|  | No | 23(59.0) | 6(40.0) | 6(60.0) | 0.430 |
|  | Yes | 16(41.0) | 9(60.0) | 4(40.0) |  |
| Endocrine therapy | | | | | |
|  | No | 22(56.4) | 13(86.7) | 6(60.0) | 0.117 |
|  | Yes | 17(43.6) | 2(13.3) | 4(40.0) |  |
| DFI (month) | | | | | |
|  | ≤12 | 12(30.8) | 3(20.0) | 1(10.0) | 0.672 |
|  | >12 | 20(51.3) | 8(53.3) | 6(60.0) |  |
|  | De novo IV stage | 7(17.9) | 4(26.7) | 3(30.0) |  |
| Previous trastuzumab treatment | | | | | |
|  | Neoadjuvant setting | 5(12.8) | 0(0) | 0(0) | 0.324 |
|  | Adjuvant setting | 16(41.0) | 5(33.3) | 1(10.0) |  |
|  | Metastatic setting | 31(79.5) | 11(73.3) | 10(100.0) |  |
| Previous anti‐HER2 drugs | | | | | |
|  | Pyrotinib | 24(61.5) | 8(53.3) | 9(90.0) | 0.895 |
|  | Pertuzumab | 5(12.8) | 2(13.3) | 3(30.0) |  |
|  | TDM-1 | 1(2.6) | 0(0) | 1(10.0) |  |
|  | Aptinib | 1(2.6) | 0(0) | 1(10.0) |  |
|  | Lapatinib | 8(20.5) | 3(20.0) | 1(10.0) |  |
|  | Anlotinib | 1(2.6) | 0(0) | 0(0) |  |
| Number of sites in primary recurrence | | | | | |
|  | 1 | 19(48.7) | 10(66.7) | 5(50.0) | 0.497 |
|  | >1 | 20(51.3) | 5(33.3) | 5(50.0) |  |
| Lines of inetetamab in metastatic setting | | | | | |
|  | 1 | 2(5.1) | 1(6.7) | 0(0) | 0.507 |
|  | 2 | 12(30.8) | 7(46.7) | 2(20.0) |  |
|  | ≥3 | 25(64.1) | 7(46.7) | 8(80.0) |  |
| Number of sites before inetetamab | | | | | |
|  | 1 | 13(33.3) | 5(33.3) | 3(30.0) | 0.696 |
|  | 2 | 6(15.4) | 3(20.0) | 0(0) |  |
|  | ≥3 | 20(51.3) | 7(46.7) | 7(70.0) |  |
| Metastatic sites before inetetamab | | | | | |
|  | Local sites | 12(30.8) | 7(46.7) | 4(40.0) | 0.388 |
|  | Lymph node | 19(48.7) | 7(46.7) | 5(50.0) |  |
|  | Bone | 14(35.9) | 1(6.7) | 6(60.0) |  |
|  | Brain | 6(15.4) | 3(20.0) | 4(40.0) |  |
|  | Lung | 12(30.8) | 2(13.3) | 1(10.0) |  |
|  | Liver | 13(33.3) | 3(20.0) | 5(50.0) |  |
|  | Others | 8(20.5) | 2(13.3) | 0(0) |  |
| Visceral metastases | | | | | |
|  | Yes | 22(56.4) | 5(33.3) | 5(50.0) | 0.277 |
|  | No | 17(43.6) | 10(66.7) | 5(50.0) |  |

Supplementary Table 3 Patients treated with different combined regimens

| Characteristics | | Patients, No (%) | | | p-value |
| --- | --- | --- | --- | --- | --- |
|  |  | Inetetamab+vinorelbine +pyrotinib, N=31(48.4) | Inetetamab+abraxane +pyrotinib, N=7(10.9) | Inetetamab+other therapeutic agents, N=26(40.6) |  |
| Age, median (range years) | | 46(27-58) | 49(39-64) | 43.5(29-67) | 0.256 |
| Menstrual status | | | | | |
|  | pre | 19(61.3) | 3(42.9) | 16(61.5) | 0.625 |
|  | post | 12(38.7) | 4(57.1) | 10(38.5) |  |
| ECOG performance status | | | | | |
|  | 0-1 | 27(87.1) | 4(57.1) | 21(80.8) | 0.209 |
|  | ≥2 | 4(12.9) | 3(42.9) | 5(19.2) |  |
| Pathological type | | | | | |
|  | Invasive ductal cancer | 31(100) | 6(85.7) | 25(96.2) | 0.101 |
|  | Invasive lobular cancer | 0(0) | 1(14.3) | 1(3.8) |  |
| HER2 expression | | | | | |
|  | IHC2+ and FISH+ | 6(19.4) | 1(14.3) | 6(23.1) | 0.908 |
|  | IHC3+ | 25(80.6) | 6(85.7) | 20(76.9) |  |
| HR status at metastatic setting | | | | | |
|  | Positive | 14(45.2) | 3(42.9) | 12(46.2) | 1.000 |
|  | Negative | 17(54.8) | 4(57.1) | 14(53.8) |  |
| Surgery | | | | | |
|  | No | 4(12.9) | 2(28.6) | 4(15.4) | 0.552 |
|  | Yes | 27(87.1) | 5(71.4) | 22(84.6) |  |
| Radiotherapy | | | | | |
|  | No | 17(54.8) | 4(57.1) | 14(53.8) | 1.000 |
|  | Yes | 14(45.2) | 3(42.9) | 12(46.2) |  |
| Endocrine therapy | | | | | |
|  | No | 18(58.1) | 6(85.7) | 17(65.4) | 0.404 |
|  | Yes | 13(41.9) | 1(14.3) | 9(34.6) |  |
| DFI (month) | | | | | |
|  | ≤12 | 10(32.3) | 2(28.6) | 4(15.4) | 0.623 |
|  | >12 | 15(48.4) | 3(42.9) | 16(61.5) |  |
|  | De novo IV stage | 6(19.4) | 2(28.6) | 6(23.1) |  |
| Previous trastuzumab treatment | | | | | |
|  | Neoadjuvant setting | 4(12.9) | 0(0) | 1(3.8) | 0.677 |
|  | Adjuvant setting | 16(51.6) | 3(42.9) | 8(30.8) |  |
|  | Metastatic setting | 23(74.2) | 5(71.4) | 20(76.9) |  |
| Previous anti‐HER2 drugs | | | | | |
|  | Pyrotinib | 19(61.3) | 2(28.6) | 20(76.9) | 0.786 |
|  | Pertuzumab | 4(12.9) | 2(28.6) | 4(15.4) |  |
|  | TDM-1 | 1(3.2) | 0(0) | 1(3.8) |  |
|  | Aptinib | 1(3.2) | 0(0) | 1(3.8) |  |
|  | Lapatinib | 6(19.4) | 2(28.6) | 4(15.4) |  |
|  | Anlotinib | 0(0) | 0(0) | 1(3.8) |  |
| Number of sites in primary recurrence | | | | | |
|  | 1 | 18(58.1) | 5(71.4) | 11(42.3) | 0.304 |
|  | >1 | 13(41.9) | 2(28.6) | 15(57.8) |  |
| Lines of inetetamab in metastatic setting | | | | | |
|  | 1 | 2(6.5) | 1(14.3) | 0(0) | 0.153 |
|  | 2 | 9(29.0) | 4(57.1) | 8(30.8) |  |
|  | ≥3 | 20(64.5) | 2(28.6) | 18(69.2) |  |
| Number of sites before inetetamab | | | | | |
|  | 1 | 11(35.5) | 3(42.9) | 7(26.9) | 0.356 |
|  | 2 | 5(16.1) | 1(14.3) | 1(3.8) |  |
|  | ≥3 | 15(48.4) | 3(42.9) | 18(69.2) |  |
| Metastatic sites before inetetamab | | | | | |
|  | Local sites | 11(35.5) | 3(42.9) | 9(34.6) | 0.741 |
|  | Lymph node | 15(48.4) | 3(42.9) | 13(50.0) |  |
|  | Bone | 9(29.0) | 0(0) | 12(46.2) |  |
|  | Brain | 6(19.4) | 1(14.3) | 6(23.1) |  |
|  | Lung | 9(29.0) | 2(28.6) | 4(15.4) |  |
|  | Liver | 12(38.7) | 3(42.9) | 6(23.1) |  |
|  | Others | 7(22.6) | 0(0) | 4(15.4) |  |
| Visceral metastases | | | | | |
|  | Yes | 18(58.1) | 4(57.1) | 10(38.5) | 0.304 |
|  | No | 13(41.9) | 3(42.9) | 16(61.5) |  |
